# Biomarker *δ*^13^C values record consistent savanna vegetation and variable alkalinity of Lake Olduvai during Pleistocene wet/dry cycles

**DOI:** 10.1073/pnas.2508896122

**Published:** 2025-12-29

**Authors:** Kelsey E. Doiron, Devon E. Colcord, Andrea M. Shilling, Jackson K. Njau, Ian G. Stanistreet, Harald Stollhofen, Kathy D. Schick, Nicholas P. Toth, Simon C. Brassell

**Affiliations:** ^a^Department of Earth & Atmospheric Sciences, Indiana University, Bloomington, IN 47401; ^b^Department of Earth, Ocean, and Ecological Sciences, University of Liverpool, Liverpool L69 3BX, United Kingdom; ^c^The Stone Age Institute, Gosport, IN 47433; ^d^GeoZentrum Nordbayern, Friedrich-Alexander-Universität, Erlangen 91054, Germany

**Keywords:** plant wax, carbon isotopes, alkenones, steroids, hopanoids

## Abstract

Climate records for the early Pleistocene in eastern Africa aid reconstruction of the ecology and landscapes occupied by hominins. Environmental changes associated with alternating wet/dry cycles during this critical interval in hominin evolution are recorded in isotopic profiles from lake sediments from Olduvai Gorge. The Olduvai Gorge Coring Project targeted the depocenter of Paleolake Olduvai, recovering a stratigraphic succession between the Bed I Basalt (~1.90 Ma) and Tuff IF (~1.80 Ma) datums that span hominin fossil horizons in nearby outcrops. Lacustrine claystones from the lower part of this interval are characterized by high C_org_ (avg. 2.5 %). They display variations in *δ*^13^C_org_ reflecting temporal changes in organic matter (OM) sources. Stratigraphic profiles for *n*-alkane *δ*^13^C values derived from plant waxes are consistent throughout wet/dry cycles indicating stability in savanna vegetation, refuting evidence for increased C_4_ grasses during drier intervals. *δ*^13^C values for *n*-alkanes derived from aquatic macrophytes and for algal biomarkers increase during drier episodes, reflecting use of bicarbonate as a carbon source triggered by enhanced lake alkalinity. *δ*^13^C_org_ profiles reflect variations in inputs of terrestrial vs. aquatic OM during wet/dry cycles, which are mirrored by *δ*^13^C values for hop-17(21)-ene derived from heterotrophic bacteria utilizing sedimentary OM. The *δ*^13^C records from Paleolake Olduvai sediments show temporal changes in OM sources rather than shifts in the proportion of C_3_ and C_4_ vegetation, indicating that precession-driven climate cycles primarily affected lake environments and associated aquatic resources. Thus, intervals of high climate variability did not necessarily cause changes in savanna vegetation affecting hominin habitats.

## Significance

Fossil and archaeological records confirm that the lacustrine setting of Olduvai Gorge was a sustained habitat for hominins in the early Pleistocene. Sediments deposited in Paleolake Olduvai spanning forty thousand years record evidence of climatic and environmental changes and episodes of ecological stability in the landscapes occupied by hominins. Isotopic signatures diagnostic of plant waxes document consistency in savanna vegetation during a succession of wet/dry climate cycles, whereas those associated with aquatic biota attest to increased lake alkalinity during drier intervals. Hominins would have experienced a stable terrestrial ecosystem with a consistent mix of woodland and grassland, while water resources were affected by pronounced temporal variations in the hydrological cycle regulating the size and chemistry of Paleolake Olduvai.

**Fig. 1.**
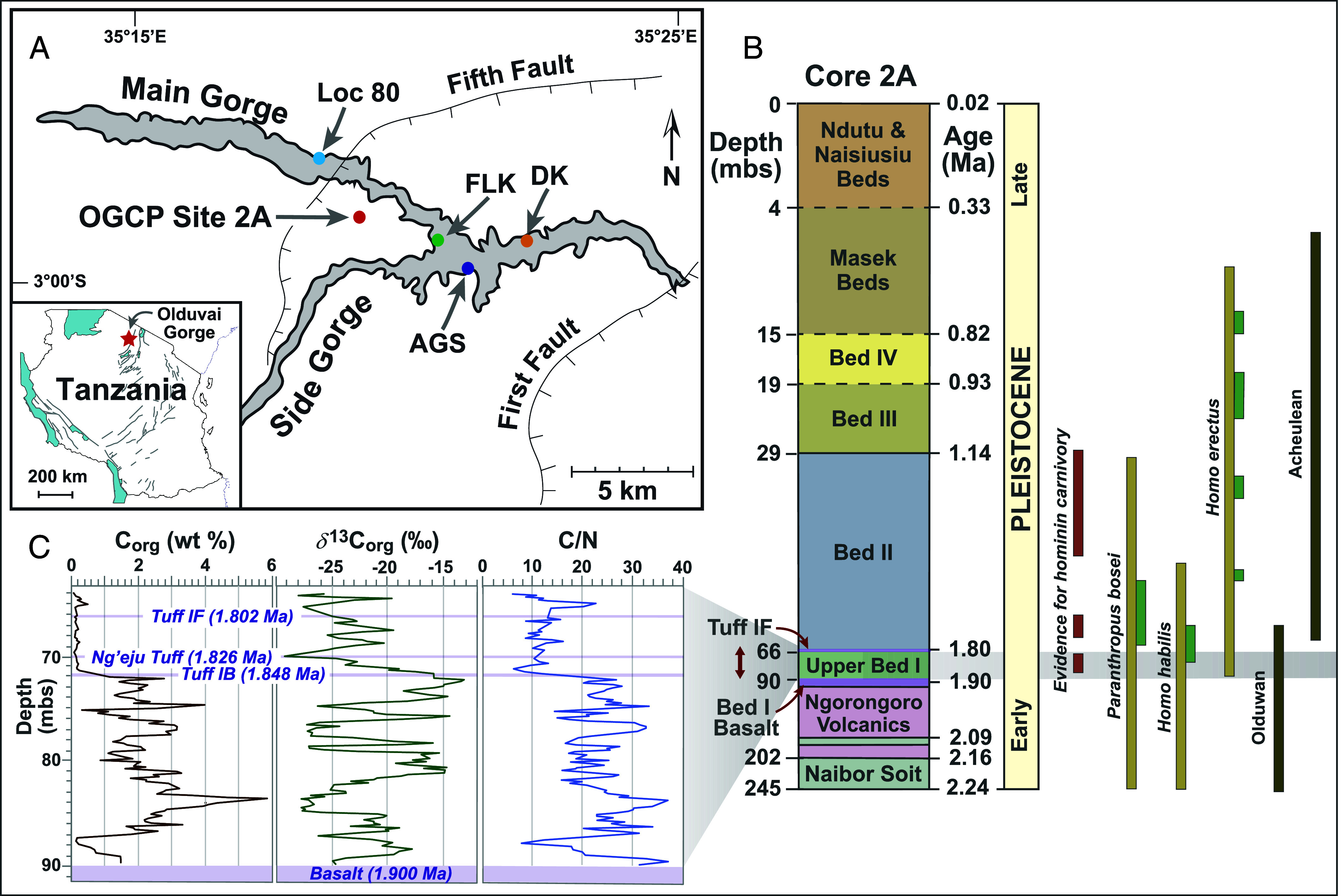
(*A*) Location of Olduvai Gorge in northern Tanzania showing the site of Olduvai Gorge Coring Project (OGCP) Core 2A and the locations of other key studies [Loc 80 (7); FLK (15); DK (16, 31); AGS (17)]. (*B*) Chronostratigraphic column for the Core 2A succession (mbs = meters below surface) that includes ages for the boundaries of each stratigraphic unit [(3); *SI Appendix*, Table S1] and shows the position of Upper Bed I between the Bed I basalt and Tuff IF (10) and approximate temporal ranges for evidence of hominin carnivory at Olduvai Gorge based on evidence from taphonomy and stone tool marks on fossil bones [red bars; (17–19)], hominin species in eastern Africa [taupe bars; (32, 33)] and fossil hominins at Olduvai Gorge [green bars; (34–44)], and hominin stone technologies [brown bars; (20–22)]. This compilation places *H. ergaster* with *H. erectus* (cf. 26). (*C*). A compilation of stratigraphic profiles for biogeochemical data (% C_org_,‰ *δ*^13^C_org_, and C/N ratios) for the Upper Bed I sequence (45–49) noting ages for the marker beds (49). % C_org_ and C/N ratios show a marked decrease above Tuff IB associated with biomarker evidence for enhanced microbial alteration of OM (47). Data for the interval from the Bed I basalt to Tuff IB are included in Dataset S1.

High-resolution paleoenvironmental reconstructions can provide a temporal and spatial context to evaluate connections between climate-driven environmental changes and evolutionary advances. For localities associated with sparse anthropological records (e.g., rare and incomplete hominin fossils) these studies both complement and augment evidence from sediment geochemistry, stone artifacts, vertebrate fossils, and plant assemblages (1). Olduvai Gorge is a world-famous hominin fossil site adjacent to the Great Rift Valley of Tanzania that gained fame from high-profile discoveries of hominin fossils and stone tools with excellent age control founded on a high-resolution tephrochronological framework (2, 3). Anthropologists and geologists have conducted extensive studies of the ~2-My sequence of fluvial, lacustrine, and volcanic sediments exposed in outcrops to evaluate the potential role of climate change in hominin evolution (1, 2, 4–9). The Olduvai Gorge Coring Project (OGCP; Fig. 1 and *SI Appendix*) recovered this stratigraphic succession and extended it to ~2.5 Ma (10). The succession of lacustrine sediments above the Bed I basalt lava at Olduvai Gorge, designated as Upper Bed I [~1.90 to 1.80 Ma; Fig. 1*B* (3); *SI Appendix*], correlates with the peak of hominin diversity in eastern Africa (11, 12), an increase in cranial capacity (13, 14), and occurrences of stone tools and butchered faunal remains associated with hominin activities [(15–22); Fig. 1*B*]. Fossil and archaeological records confirm that the lacustrine setting of Olduvai Gorge was a sustained habitat for hominins and vertebrates in the early Pleistocene, which underscores the importance of characterizing the timing and scale of environmental changes and episodes of ecological stability at this location. Salinity data suggest that precipitation during October, November, and December (“short rains”) influenced Paleolake Olduvai during Upper Bed I deposition, reflecting the intensity of the southeasterly monsoon and southern hemisphere insolation (23). Global climate records indicate that this time interval coincides with intensification of Walker Circulation (11, 24–28) which affected rainfall over eastern Africa (29) and led to increasing aridity from 2.11 to 1.66 Ma, as recorded by paleosols (30). However, evidence for aridification from lacustrine sediments occurs toward the end of this time interval following wetter episodes recorded by enhanced lake levels and size (11).

**Fig. 2.**
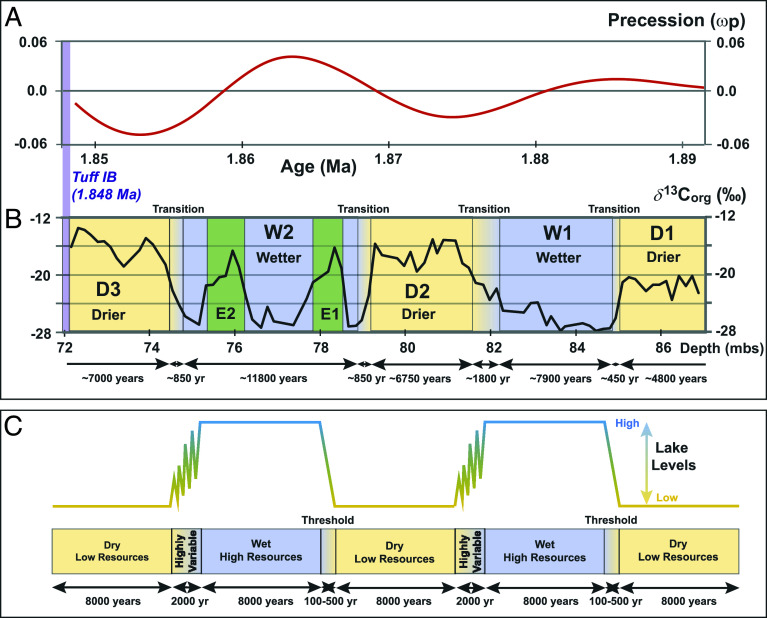
(*A*) Temporal variation in precession (adapted from ref. 46) during Upper Bed I interval. (*B*) *δ*^13^C_org_ values for Upper Bed I from OGCP Core 2A portraying the inferred alternating sequence of wetter (avg. –24.13‰) and drier (avg. –17.85‰) intervals (10, 45–48, 50) and transitions between them (avg. –22.69‰) based their timing and duration based on the interpolated chronology (*SI Appendix*). (*C*) Schema describing the influence of eccentricity-modulation of precession on eastern African lake levels (after ref. 52), illustrating a similarity with the rhythms of wet/dry cycles in the stratigraphic record for *δ*^13^C_org_ from Upper Bed I (*B*; Dataset S1). However, the timing of wet/dry cycles relative to the phase of precession differs among eastern African lakes (e.g., refs. 7, 8, 45, 54, and 55). Fluctuations in lake levels are only depicted during regressions (52) but likely also occurred during transgressions and throughout each precession cycle. However, the temporal resolution of sampling of OGCP sediments (*SI Appendix*) precludes recognition of such fluctuations, which may also exert minimal influence on sedimentary OM in the lake depocenter.

Prior investigations of the sedimentary succession formed in Paleolake Olduvai indicate temporal changes in its extent and depth linked to variations in solar insolation modulated by orbital precession (1, 10, 31, 50). They suggest that the lake reached its maximum size and depth during Upper Bed I. The succession of lacustrine claystones deposited at this time coincides with a substantive decrease in the supply of volcanic sediment from the surrounding Ngorongoro Volcanic Highlands (1, 10). Environmental reconstructions for Upper Bed I at Olduvai Gorge have documented changes in vegetation and effective moisture associated with wet/dry cycles [Fig. 2 (7, 45–48, 51)] within a long-term aridification trend that persists into the modern climate regime of eastern Africa (5, 52, 53). Previous studies from outcrops and cores (7, 45) correlated precession-driven cyclicity in solar insolation with variations in *δ*^13^C_org_ (Fig. 2), which were attributed to changes in vegetation from a C_3_ dominated landscape during wetter intervals to a C_4_ graminoid-dominated environment during the dry phase of wet/dry cycles. Marked variations in climate and vegetation are thought to have influenced hominin diversity, dispersal, and evolution (33), including the emergence of *Homo* (7).

Variations in effective moisture during deposition of Upper Bed I are also reflected in the *δ*^2^H variations of terrestrial plant waxes (45–48). Plant remains (macrofossils and phytoliths) in the lake sediments record a vegetation dominated by palms, typha, and sedges (56), whereas occurrences of diatoms, chrysophyte cysts, sponge spicules, and diagnostic sponge biomarkers indicate the presence of wetlands and freshwater (groundwater or river) springs within the lake catchment (46, 47, 57, 58). Paleolake Olduvai was a saline/alkaline system within a largely closed basin that experienced substantial changes in its hydroclimate. Lower lake levels during drier conditions led to shifts in sedimentary facies, decreased areal extent, and an increase in saline/alkaline conditions (1, 23, 31, 59–64), reflected in mineral assemblages, notably the presence of diagenetic analcime (65, 66), plus multiple instances of complete lake desiccation (25, 43). Elevated levels for Paleolake Olduvai (48) occur during an interval of the early Pleistocene (1.9 to 1.7 Ma) when climatic variability led to an expansion of lakes in eastern Africa correlated by integrated magnetostratigraphy and tephrochronology (11, 24, 27, 52). At Olduvai, wetter intervals correspond with precession maxima [Fig. 2; (7, 45)], whereas drier intervals, most notably lake desiccation during emplacement of Tuff IF, occur during precession minima (47, 66). Thus, the phasing of precession linked to monsoonal precipitation in the early Pleistocene at Olduvai differs from other locations in eastern Africa (54, 55, 67–69). This difference may reflect the concomitant shift from southern to northern hemispherical influence on ice volume and its timing and intensification in the early Pleistocene (68–70) plus the propensity for the expression of local variations in the connection between precession and precipitation in equatorial latitudes (71, 72).

The Upper Bed I interval from the OGCP sediment cores recovered in 2014 provides samples with better physical integrity than those from outcrops (7, 10) and are characterized by superior preservation of biomarkers (45–48, 51). They afford the opportunity to garner robust paleoclimate proxy data to assess temporal features in the *δ*^13^C profiles for OM, terrestrial vegetation, and aquatic producers over wet/dry cycles. In particular, they enable an assessment of *δ*^13^C profiles for algal and bacterial biomarkers, expanding on prior isotope studies for the Early Pleistocene in Olduvai Gorge and eastern Africa that have focused on pedogenic carbonates (60, 73), rhizoliths (74), paleosol horizons (75, 76), *n*-alkanes representative of terrestrial vegetation and aquatic macrophytes (7, 15, 17, 77), and their *δ*^2^H values of *n*-alkanes reflecting precipitation (15, 45, 46, 48, 78).

Here we demonstrate that evaluation of a broader base of biomarkers besides *n*-alkanes allows assessment of connections between the *δ*^13^C signatures of diverse sources of OM and sedimentary *δ*^13^C_org_ values, including the possibility that variations in the latter do not simply reflect shifts in relative contributions from C_3_ and C_4_ plants. We also show that examination of bacterial biomarkers helps distinguish their role as sources of OM and in its preservation, whereas algal biomarkers provide evidence for changes in lake alkalinity. Thus, biomarker isotopic compositions facilitate assessment of the balance between OM contributions from algae and higher plants in wetter and drier intervals, providing a more comprehensive evaluation than bulk geochemical assessments combining *δ*^13^C_org_ and C/N ratios (79). Overall, such datasets assist in reconstruction of both vegetation and lake ecology based on *δ*^13^C records of lacustrine sediments, advancing understanding of environments inhabited by hominins in eastern Africa.

## Results

### Carbon Isotope Profiles of Early Pleistocene Wet/Dry Cycles in Olduvai Gorge.

**Fig. 3.**
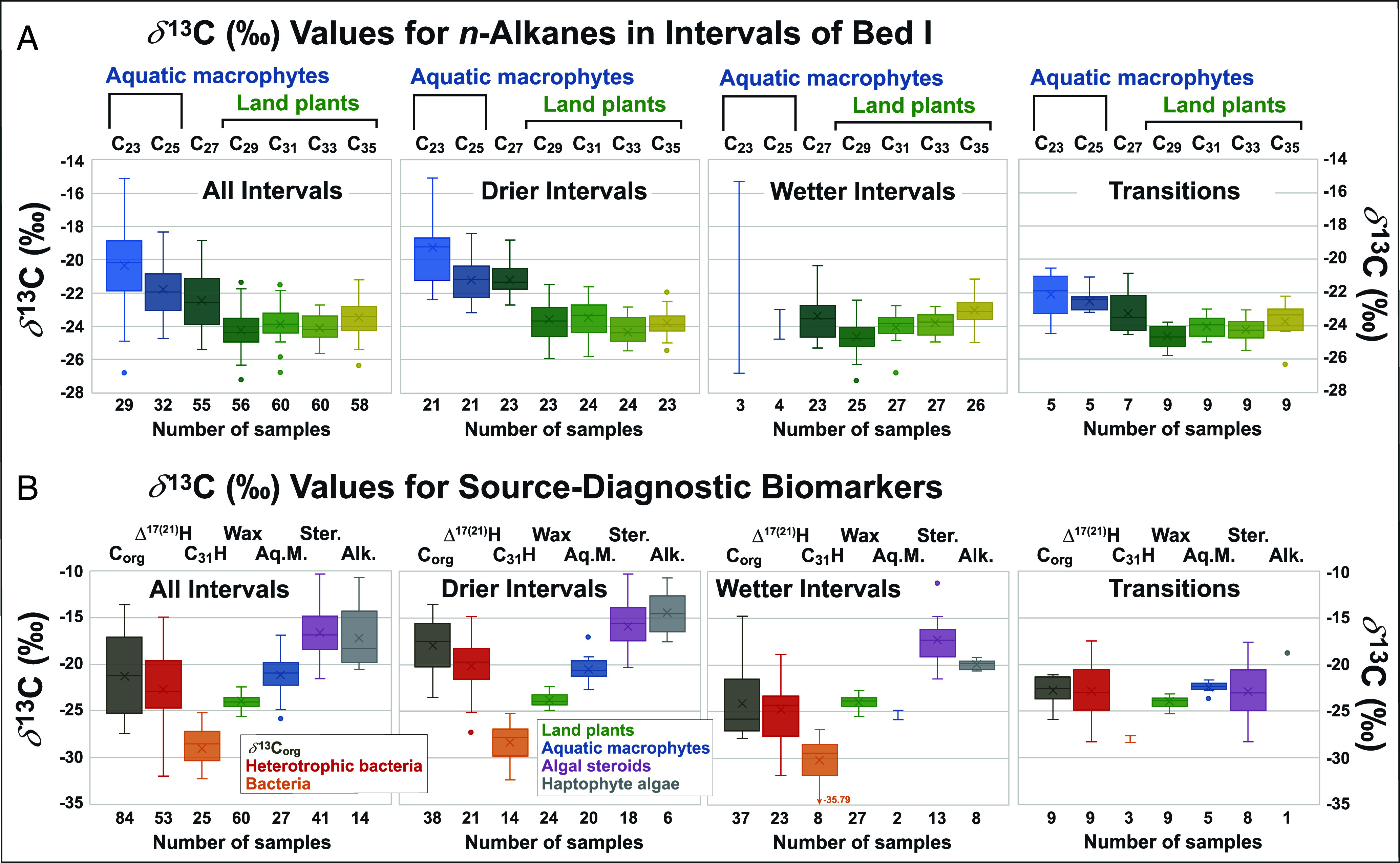
Box-and-whisker plots of *δ*^13^C data for source-specific biomarkers within the sequence of wet/dry cycles of Bed I of OGCP Core 2A (Data from Dataset S1). The three series of plots reflect data representing the entire sequence, drier intervals, and wetter intervals, respectively. (*A*) Individual *n*-alkanes sourced from aquatic macrophytes (C_23_ and C_25_) show enrichment in *δ*^13^C relative to those from land plants (C_29_, C_31_, C_33_, and C_35_). Also, *δ*^13^C ranges for *n*-alkane homologs from land plants are temporally coherent, varying little between drier and wetter intervals. (*B*) *δ*^13^C ranges for source-specific biomarkers reflect their origins. Abbreviations: C_org_ = *δ*^13^C_org_, Δ^17(21)^H = hop-17(21)-ene, C_31_H = 17β(H),21β(H)-homohopane (C_31_), Wax = land plants *n*-alkanes (C_29_, C_31_, C_33_, and C_35_), Aq.M. = aquatic macrophyte *n*-alkanes (C_23_ and C_25_), Ster. = algal steroids, Alk. = alkenones. Δ^17(21)^-Hopene differs from the ββ-hopanes and tracks *δ*^13^C_org_, consistent with an origin from heterotrophic bacteria. The *δ*^13^C range for land plants *n*-alkanes is narrow, aligning *δ*^13^C_org_ in wetter intervals. Algal constituents exhibit a marked enrichment of *δ*^13^C in drier intervals, likely reflecting use of bicarbonate for their carbon source as lake alkalinity increased.

Initial organic geochemical studies of Core 2A sediments evaluated C_org_ contents, *δ*^13^C_org_, and C/N ratios (Fig. 1*C*) and biomarker compositions (45–48). C_org_ contents and C/N ratios decline above Tuff IB (Fig. 1*C*), the first major eruptive unit derived from Olmoti volcano, associated with the progressive shallowing, oxidation, and desiccation of Paleolake Olduvai (47, 48), prompting a focus on the lower part of Upper Bed I. This interval from the Bed I basalt to Tuff IB (86.9 to 72.3 mbs) consists of lacustrine claystones interbedded with sandy claystones and is characterized by high C_org_ contents (avg. 2.5 %; Fig. 1*C*), including several laminated intervals that yield high γ-ray signals (48). The absence of both phytoliths and diatoms is consistent with other evidence for enhanced salinity/alkalinity, in contrast to the earlier lacustrine interval within the Naibor Soit Formation (56, 65). The *δ*^13^C_org_ profile for this section comprises five intervals (designated D1, W1, D2, W2, and D3) reflecting alternating wet–dry conditions [Fig. 2*B*, (45–48)] related to precession cycles [Fig. 2*A*, (48)]. The stratigraphic profile for *δ*^13^C_org_ correlates with an independent set of sample-matched elemental data (Mg, Al, Ti) determined by scanning XRF analysis conducted at 10 mm stratigraphic resolution (66). The values for *δ*^13^C_org_ through the Upper Bed I sequence (Dataset S1) range from −13.5‰ to −27.8‰ (avg. = −21.1‰; Figs. 2*B* and 3*B*) but with a pronounced difference between their interquartile values for drier and wetter intervals of −15.6‰ to −20.2‰ vs. −21.4‰ to −27.1‰, respectively, and a 6‰ in their averages [avg. = −17.8‰ vs. −24.1‰; (48)]. The difference in *δ*^13^C_org_ values for drier and wetter intervals are statistically significant (*SI Appendix*, Fig. S1) and parallel those recorded for Upper Bed I sediments sampled at low resolution (7) from outcrops at Location 80 (Fig. 1*A*). These data have been interpreted to represent changes in the dominant source of sedimentary OM, reflecting landscape shifts from a prominence of C_3_ woodland plants during wetter intervals to C_4_ grasses during drier phases (7).

### Biomarker δ13C Compositions.

Prior studies of the composition of terrestrial and aquatic biomarkers in the Upper Bed I section from OGCP Core 2A show variations in the abundances of diagnostic compounds that parallel the cycles in *δ*^13^C_org_ values and confirm higher relative amounts of aquatic lipids (e.g., alkenones) during drier intervals and plant waxes (e.g., C_31_ and C_33_ *n*-alkanes) during wetter intervals (45–48). Investigations of sediments from outcrops and cores from Olduvai Gorge have also explored *δ*^13^C and *δ*^2^H profiles for plant wax *n*-alkanes to assess connections between temporal changes in climate and paleolandscape to provide contextual evidence for the dynamic habitat experienced by hominins (7, 45, 47, 48). The *δ*^13^C values for *n*-alkanes from low-resolution sampling of outcrop materials distinguished contributions from algae, aquatic macrophytes, and land plants with variations in *δ*^13^C_org_ attributed to a shift in the relative contributions of C_3_ and C_4_ plants (7). However, the *δ*^13^C data for *n*-alkanes in this prior study derived from only 1 to 4 samples from individual wetter and drier intervals (7). The sediment sequence from OGCP Core 2A afforded the opportunity to explore such records at markedly higher temporal resolution and augment analyses of *n*-alkanes with examination of *δ*^13^C profiles for suites of algal and microbial biomarkers. Thus, the comprehensive assessment of the *δ*^13^C profiles for a series of source-specific terrestrial and aquatic biomarkers through the sedimentary succession of OGCP Core 2A prior to Tuff IB deposition expands on previous biogeochemical studies (10, 45–49, 51) and captures the simultaneous responses of terrestrial vegetation and aquatic biota to short-term climate changes and hydroclimate shifts during this time interval. Box-and-whisker plots portray the differences among the *δ*^13^C values for terrestrial and aquatic biomarkers (Dataset S1) derived from different source organisms and, in some instances, illustrate systematic variability between wetter and drier intervals (Fig. 3), as confirmed by statistical analysis (*SI Appendix*, Fig. S1).

## Discussion

### Plant Waxes and Aquatic Macrophytes.

The ranges for individual *n*-alkanes show a stark difference between homologs derived from aquatic macrophytes (C_23_, C_25_) and land plant waxes (C_29_, C_31_, C_33_, C_35_; Fig. 3*A*). The latter suite of *n*-alkane *δ*^13^C values derived from terrestrial vegetation reflect the combined contributions from a complex mixture of C_3_ and C_4_ plants but record a dominance of inputs from C_4_ plants (>60 %) throughout the stratigraphic section based on their average *δ*^13^C values [−23.88‰, (67)]. These data also indicate minimal differences among the collective average for plant wax *n*-alkanes between wetter and drier intervals (−23.89‰ and −23.77‰, respectively) and the transitions between them (−24.13‰; Dataset S1 and *SI Appendix*, Fig. S1). Among leaf wax *n*-alkanes, *n*-C_31_ is representative of contributions from both C_3_ and C_4_ plants and its *δ*^13^C values should be sensitive to any change in their relative proportions (80) associated with transitions from drier to wetter conditions and vice versa. However, the *δ*^13^C values for *n*-C_31_, like other *n*-alkane homologs, vary little throughout the entire Upper Bed I sequence, with an average of –23.79‰ and a SD of 0.88. Studies of contemporary terrestrial vegetation indicate that *n*-C_33_ can serve as a better representative of grass contributions and therefore reflect temporal shifts in the proportion of C_3_ vs. C_4_ grasses (78). However, the *δ*^13^C values for *n*-C_33_, like those for *n*-C_31_, show minimal variations throughout the sequence of wet/dry cycles in Upper Bed I. There is minor ^13^C enrichment in the *δ*^13^C values for *n*-C_35_ relative to *n-*C_33_ (Fig. 3*A*; averages = −23.48‰ and −24.10‰, respectively), yet the difference between the two *n*-alkane homologs is similar for wetter and drier intervals and the transitions between them. The *δ*^13^C values for *n*-alkanes in wetter intervals show progressive ^13^C enrichment from C_29_ to C_31_ to C_33_ to C_35_ (averages = −24.62‰, −24.04‰, −23.89‰, and −23.08‰, respectively; Dataset S1). Thus, the *n*-alkane *δ*^13^C values do exhibit a limited tendency toward greater ^13^C enrichment for higher homologs, which are more abundant in grasses (78, 80, 81). Overall, the *δ*^13^C results for *n*-alkanes confirm a temporal consistency in sediment inputs of plant waxes that suggests minimal change in terrestrial vegetation, especially the proportion of C_4_ plants, from wetter to drier intervals. Based on modern analogs, *n*-C_27_ and *n*-C_29_ are generally considered to be representative of woody angiosperm contributions in most environments (82–85), yet for Upper Bed I their *δ*^13^C values are also broadly similar throughout the sequence of wet/dry cycles, which further suggests a muted response in the vegetation within the surrounding catchment to the precession-driven climate cycles. Indeed, the stratigraphic consistency of their values provides no evidence for significant changes in contributions from C_4_ plants during drier intervals as previously suggested, based on outcrop material from Location 80 (Fig. 1*A*) for the same stratigraphic interval (7). Our data reveal a temporal consistency in contributions from C_4_ plants, both grasses (78, 79, 81) and sedges (86–88). They also preclude attributing shifts in *n*-alkane *δ*^13^C values to contributions from papyrus, a C_4_ freshwater plant dominated by *n*-C_31_ with a *δ*^13^C value of ~−20‰ (89). Thus, the evidence for sustained consistency in the relative contributions of C_3_ and C_4_ plants, means that temporal shifts in *δ*^13^C_org_ reflect changes in sources of OM rather than variations in the proportion of woodland and grassland vegetation as previously proposed (7). Specifically, differences in *δ*^13^C_org_ would therefore reflect transitions in the dominant source of OM from terrestrial vegetation during wetter intervals to aquatic producers during drier intervals, which can be assessed by consideration of the *δ*^13^C profiles for aquatic biomarkers.

The range of *δ*^13^C values (–21.12‰ to –26.29‰) for plant waxes aligns closely with the *δ*^13^C_org_ of bulk OM during wetter intervals, whereas the *δ*^13^C for *n*-alkanes derived from aquatic macrophytes (90), especially *n*-C_23_, are higher (avg. –21.11‰). Unlike the consistent values for *n*-alkanes (i.e., *n*-C_29_, *n*-C_31_, *n*-C_33_, and *n*-C_35_) derived from terrestrial plants captured in the sediments from OCGP Core 2A, *δ*^13^C values of the *n*-alkanes (primarily *n*-C_23_, but also *n*-C_25_) representative of contributions from aquatic macrophytes show evidence for ^13^C enrichment during lake regression (i.e., drier intervals) associated with increased salinity and alkalinity (Fig. 3), notwithstanding the limited dataset for these *n*-alkanes during wetter intervals due to their low concentrations. All three photosynthetic pathways (C_3_, C_4_, and CAM) are found in modern aquatic plants but in alkaline waters, submerged macrophytes can also assimilate bicarbonate, yielding *δ*^13^C isotopic signatures comparable with C_4_ processes (91, 92). Thus, the ^13^C enrichment observed in the *n*-alkanes derived from submerged aquatic macrophytes likely results from increased lake alkalinity during drier intervals triggering an uptake of bicarbonate by these plants, influencing the observed *δ*^13^C values for the shorter-chain *n*-alkanes.

### Bacterial Signatures.

**Fig. 4.**
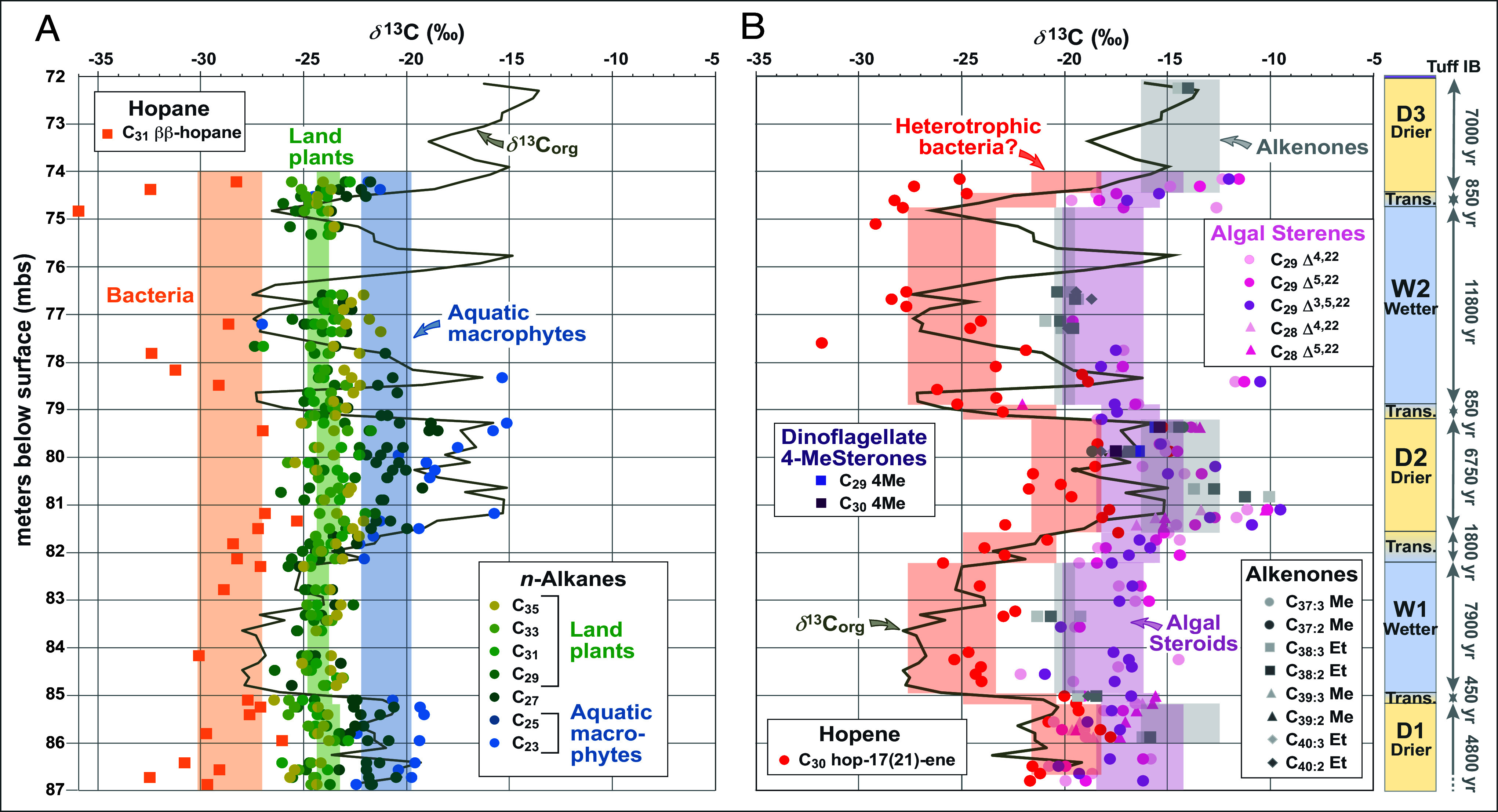
(*A*) Stratigraphic profiles from 87 mbs (3.3 m above the Bed I basalt) to Tuff IB (72 mbs) for *δ*^13^C_org_ and for source-specific biomarkers: *n*-alkanes from land plants (C_29_, C_31_, C_33_, and C_35_) and aquatic macrophytes (C_23_ and C_25_) and ββ-hopanes from bacteria (Data from Dataset S1). Both land plant *n*-alkanes and ββ-hopanes exhibit a coherent range of *δ*^13^C values throughout the sediment sequence (cf. Fig. 3), represented by green and orange shading, respectively, and show no correlation with the temporal shifts in *δ*^13^C_org_. *n*-Alkanes from aquatic macrophytes (blue shading), reflecting both submerged and partially submerged plants, show increased enrichment in ^13^C (higher *δ*^13^C) during the drier intervals (D1 and D2). (*B*) Stratigraphic profiles for *δ*^13^C_org_, for steroidal biomarkers (sterenes and steroidal ketones) and alkenones, both representative of algal sources, showing the variation in their *δ*^13^C values between wetter and drier intervals (represented by lilac and blue shading, respectively; cf. Fig. 3), and for hop-17(21)-ene, which tracks *δ*^13^C_org_ through the D1 to W2 sequence (red shading; cf. Fig. 3). The correlation between *δ*^13^C_Δ17(21)H_ and *δ*^13^C_org_ (r^2^ = 0.50 excluding data from the W2/D3 transition; *SI Appendix*, Fig. S2) is consistent with the hypothesis that heterotrophic bacteria utilizing sedimentary OM as their carbon source represent the primary contributors of hop-17(21)-ene. The higher *δ*^13^C values for algal biomarkers during drier intervals are likely linked to changes in lake alkalinity leading to enhanced bicarbonate uptake by algae during lake regressions.

The differences between the *δ*^13^C profiles for *n*-alkanes derived from plant waxes vs. aquatic macrophytes are confirmed by box-and-whisker plots that also show systematic distinctions among *δ*^13^C values for biomarkers derived from other biological sources (Fig. 3*B*) further confirmed by statistical analyses (*SI Appendix*, Fig. S1). They illustrate a progressive increase in *δ*^13^C values from bacterial hopanoids to plant wax *n*-alkanes to aquatic macrophytes to algal sterenes and haptophyte alkenones. The aliphatic hydrocarbons of the Upper Bed I sediment sequence include two prominent bacterial hopanoids, ββ-homohopane and hop-17(21)-ene (46), with distinct ranges for their *δ*^13^C values (their average values differ by >6‰ (Fig. 3*B* and *SI Appendix*, Table S1) and markedly different stratigraphic profiles (Figs. 3*B* and 4). The *δ*^13^C values for ββ-homohopane are lower, i.e., more ^13^C depleted, than other biomarker constituents, consistent with the inferred decoupling of ^13^C values for bacterial hopanoids and higher plant lipids (93). They show a difference in the ranges for wetter and drier intervals (Fig. 4*A* and *SI Appendix*, Table S1), with values for the former more negative by ~2‰, albeit statistically insignificant (*SI Appendix*, Fig. S1). These data suggest a temporally consistent bacterial origin, most likely from cyanobacteria as a diagenetic product derived from bacteriohopanepolyols (Figs. 3*B* and 4*A*). Hop-17(21)-ene is often a dominant hopanoid hydrocarbon in sediments and generally assigned as a diagenetic product of hop-22(29)-ene (diploptene), which is produced by various bacteria including cyanobacteria (94–98). However, hop-17(21)-ene is also biosynthesized by bacteria and can therefore originate as a direct input to sediments rather than being formed as a diagenetic product (96). Hence, it can help discern or differentiate between potential bacterial sources given that it reflects biosynthetic pathways and processes (99). Within Upper Bed I the concentration of hop-17(21)-ene varies markedly between wet/dry cycles, in a manner unlike biomarkers derived from terrestrial vegetation and aquatic producers (46), which suggests that it likely derives from heterotrophic bacteria (93) or perhaps multiple sources, responding to environmental changes. The *δ*^13^C composition of hop-17(21)-ene (*δ*^13^C_Δ17(21)H_) changes progressively from drier intervals through transitions to wetter intervals, as reflected by average values of −20.07‰, −22.92‰, and −24.86‰. respectively. For the Upper Bed I section, the *δ*^13^C profile of hop-17(21)-ene tracks *δ*^13^C_org_ from the D1 through W2 intervals (Fig. 4*B*; r^2^ = 0.50 for 86.87 mbs to 76.60 mbs; *SI Appendix*, Fig. S2), suggesting that it records either shifts from aquatic to terrestrial sources (e.g., from cyanobacteria to acidobacteria) or, more likely, derives from heterotrophic bacteria utilizing C_org_. For the W2 to D3 transition *δ*^13^C_Δ17(21)H_ diverges from its correlation with C_org_ (r^2^ = 0.37 for D1 through D3; *SI Appendix*, Fig. S2), which suggests a possible change in the dominant bacterial source of hop-17(21)-ene associated with the drying trend. For the earlier drier intervals (D1, D2; Figs. 2*B* and 4*B*), the source of hop-17(21)-ene appears dominated by contributions from in situ bacteria, likely consuming aquatic OM, leading to its enrichment in ^13^C during episodes of elevated lake alkalinity. Within wetter intervals, sedimentary C_org_ is less enriched in ^13^C as enhanced run-off increases inputs of terrestrial OM, leading to concomitant changes in *δ*^13^C_Δ17(21)H_ of heterotrophic bacteria potentially augmented by more negative *δ*^13^C values for OM originating from aquatic producers, and/or an increase in contributions of hop-17(21)-ene from soil bacteria. The negative shift in the mean values for hop-17(21)-ene from drier to wetter intervals is antithetical to the positive shift observed in peats (93), a difference that can be attributed to the uptake of ^13^C depleted OM produced by aquatic organisms during drier intervals of elevated alkalinity.

Overall, the stratigraphic profile for *δ*^13^C_Δ17(21)H_ leads to the conclusion that hop-17(21)-ene primarily derives from heterotrophic bacteria consuming sedimentary OM. It records differences between wetter and drier intervals, reflecting the proportions of OM derived from terrestrial vegetation vs. aquatic primary producers that are preserved in the *δ*^13^C signatures of source-diagnostic biomarkers and *δ*^13^C_org_. The exception is the interval spanning the transition between W2 and D3 (75.17 mbs to 74.22 mbs; Fig. 4*B*) where the slight discordance with *δ*^13^C_org_ suggests that the source of hop-17(21)-ene may reflect more pronounced contributions as a diagenetic product of diploptene (hop-22(29)-ene) derived from photosynthetic bacteria. For the Upper Bed I sequence of Paleolake Olduvai, the profile of *δ*^13^C_Δ17(21)H_ parallels changes in the sources of sedimentary OM during wet/dry cycles (*SI Appendix*, Fig. S2). This evidence helps refine understanding of influences on the *δ*^13^C_org_ record for outcrops at Location 80 and OGCP Core 2A (Fig. 1*A*) that were previously interpreted to reflect changes in the contributions of C_3_ vs. C_4_ plants in response to hydroclimate shifts (7, 45).

### Aquatic Biomarkers and Lake Alkalinity.

Among aquatic biomarkers, the *δ*^13^C values for three compound classes were determined: sterenes, steroidal ketones, and alkenones. Alkenones are highly source-specific, deriving solely from a few species of haptophyte algae (51, 100–102). Their *δ*^13^C values therefore represent explicit signatures of photosynthetic phytoplankton. Prior work has shown that the abundances of alkenones (C_37_-C_40_ di- and triunsaturated components) and temporal variations in their unsaturation indices (U^K’^_37_, U^K’^_38_) in the Upper Bed I sediment sequence from OGCP Core 2A reflects a response to salinity/alkalinity-driven changes in Paleolake Olduvai (50). Sterols are less diagnostic of specific algae, which means that they and sterenes, formed as their diagenetic products (98, 103), serve as biomarkers representative of generic algal contributions of OM. The composition of sterenes differs from that expected from dehydration of the sterols in papyrus (104), which precludes influential contributions from this potential C_4_ source. The abundance of sterenes (C_28_ and C_29_ Δ^4,22^ and Δ^5,22^ steradienes, C_29_ Δ^3,5,22^ steratriene) in Upper Bed I sediments is markedly higher during drier intervals reflecting changes in algal productivity associated with wet/dry cycles (46, 48). Other steroidal components in the sequence include A-nor steranes (46–48) and 4-methyl-5α-stanones (98) derived from sponges and dinoflagellates, respectively. Both series of compounds are more abundant in drier intervals but determination of their *δ*^13^C values was only possible for two stanones (4,24-dimethyl-5α-cholestan-3-one, 4,23,24-trimethyl-5α-cholestan-3-one) in two samples (Fig. 4*B* and Dataset S1).

The overall interquartile ranges for the *δ*^13^C values of sterenes and alkenones in the Upper Bed I sequence are from –14.92‰ to –18.36‰ and –14.35‰ to –19.85‰, respectively (Fig. 3*B*), with excursions in *δ*^13^C values for individual sterenes to <–10‰ and >–22‰ through the sequence (Fig. 4*B* and Dataset S1). Both classes of compounds show marked differences in their *δ*^13^C values for drier and wetter intervals with interquartile ranges for sterenes of −14.12‰ to −18.37‰ (avg. = −15.82‰) vs. −16.14‰ to −19.11‰ (avg. = −17.52‰), respectively, and with intermediate values for transitional intervals (−15.28‰ to −18.07‰; avg. = −16.71‰). Alkenones show similar ranges of −12.59‰ to −16.42‰ (avg. = −14.75‰) vs. −19.50‰ to −20.52‰ (avg. = −20.33‰) for drier and wetter intervals respectively, a discrepancy in *δ*^13^C values comparable to that observed for *δ*^13^C_org_ (Fig. 3*B* and Dataset S1). The *δ*^13^C shifts in biomarkers derived from phytoplankton during drier intervals are interpreted to be driven by changes in alkalinity associated with lake regression cycles that affect the equilibrium between free CO_2_ and bicarbonate within the dissolved inorganic carbon (DIC) pool. Increases in alkalinity resulting from a significant decrease in lake volume combined with a decline in run-off and other freshwater contributions (i.e., groundwater and rivers) during drier intervals will have directly affected the DIC pool, decreasing dissolved CO_2_. This decrease in CO_2_ availability leads to enhanced uptake of bicarbonate by algae, based on modern analog studies (105). In addition, the shift in carbon source from dissolved CO_2_ to bicarbonate results in ^13^C enrichment of photosynthetic biomass causing the observed systematic difference in the *δ*^13^C values for alkenones between wetter and drier intervals and a similar, albeit of lower magnitude, feature for sterenes. Hence, the shift in *δ*^13^C values for algal biomarkers during wet/dry cycles directly reflects the influence of changes in lake alkalinity on their source phytoplankton.

Primary productivity is not constrained by high alkalinity in eastern African lakes (106), nor by low levels of dissolved CO_2_ in contemporary freshwater ecosystems (107, 108). Thus, the observation that limited availability of CO_2_ does not restrict phytoplankton growth confirms that uptake of bicarbonate can serve as the carbon source fueling photosynthesis. However, consideration of this possibility has often been overlooked in evaluation of the carbon cycle in alkaline lake systems. Evidence that CO_2_ availability does not restrict primary production also means that bicarbonate can become the dominant inorganic carbon source for algae in both shoreline and pelagic lake settings (105). The enrichment in ^13^C during drier intervals shown by the *δ*^13^C data for biomarkers associated with aquatic macrophytes (*n*-C_23_, *n*-C_25_) and algae (sterenes, alkenones) indicates that bicarbonate uptake played an influential role in facilitating continuity in primary productivity within Paleolake Olduvai during episodes of increased alkalinity. Furthermore, the effect of bicarbonate ion incorporation on biomarker *δ*^13^C values impacts secondary microbial signatures (i.e., *δ*^13^C_Δ17(21)H_) and ultimately sedimentary OM, increasing *δ*^13^C_org_ during drier intervals (Fig. 3*B*).

### Wet/Dry Climate Cycles.

The results from this study demonstrate that the pronounced temporal shifts in *δ*^13^C_org_ reflect transitions from a dominance of OM derived from terrestrial vegetation (*n*-alkanes from leaf waxes) during wetter intervals to OM from aquatic sources (*n*-alkanes from aquatic macrophytes and algal biomarkers: sterenes, 4-methylstanones, and alkenones) during drier intervals. This assessment is consistent with previous biomarker investigations of the OGCP Core 2A sediment sequence that show a decline in terrestrial contributions of OM during drier intervals, reflecting a significant decrease in inputs of terrestrial plant biomass and reduced run-off (46–48). This shift in OM sources is supported by changes in the distributions of *n*-alkanes assessed by values for TAR [terrestrial to aquatic ratio = (C_27_+C_29_+C_31_)/(C_17_+C_19_+C_21_)] and P_alg_ [proportion of algal relative to terrestrial homologs = (C_17_+C_19_)/(C_17_+C_19_+C_29_+C_31_)] (48). TAR values are markedly higher in wetter than drier intervals, whereas P_alg_ values are lower (avg. 17.5 vs. 7.0 and 0.055 vs. 0.15, respectively, for the D1 through W2 sequence). Moreover, high-resolution investigation of the *δ*^13^C compositions of a combination of terrestrial and aquatic biomarkers in ancient lacustrine sedimentary sequences aids reconstruction of temporal changes in terrestrial landscapes, paleolimnological conditions, phytoplankton dynamics, and the processes and substrates involved in carbon uptake and cycling. Overall, comprehensive suites of biomarker *δ*^13^C profiles for Upper Bed I show that variations in hydroclimate produced a rather muted response in terrestrial vegetation compared to the profound impact on carbon utilization by aquatic organisms within Paleolake Olduvai (Figs. 3 and 4). These environmental variables suggest that hominins experienced an ecological setting over ~40 ky wherein the terrestrial vegetation was stable but punctuated by marked shifts in water resources on timescales of ~6 to 12 ky. Furthermore, these results strongly suggest that changes in alkalinity need to be recognized as a potential primary driver of shifts in paleolimnologic records of sedimentary *δ*^13^C_org_, influencing carbon cycling and requiring consideration in interpretation of isotope data for paleoenvironmental reconstructions. Thus, the findings from assessment of the molecular and isotopic stratigraphic records for Upper Bed I sediments reinforce recent research insights that have emphasized why the critical role that alkalinity can play in the carbon cycle of lacustrine systems has previously been underestimated, especially its influence on phytoplankton and gross primary production (105, 107–111).

Stratigraphic profiles for biomarker *δ*^13^C values in OGCP Core 2A record detailed changes in OM sources through wet/dry climate cycles at Paleolake Olduvai and are consistent with prior studies of their distributions that recorded marked shifts in lake regression and transgression cycles (45–48). In augmenting prior studies of biomarker *δ*^13^C compositions through this stratigraphic interval, these data further validate the greater integrity of biomarker signatures in sediment cores compared to outcrop material from Olduvai Gorge, also evident in the preservation of C_org_ (49). They confirm that the sequence of wetter and drier intervals in Upper Bed I (from an interpolated age of ~1.89 Ma at 86.87 mbs to the base of Tuff IB, dated as 1.848 Ma; Fig. 2) is driven by Milankovitch precessional modulation of hydroclimate. This interval corresponds with the evidence for enhanced lake levels over eastern Africa (11, 24, 26, 51), although the response to orbital controls shows regional variations in the timing of wet/dry cycles relative to precession maxima [cf. refs. 7, 45, 54, and 55]. Variations in precession are modulated by eccentricity so that low-latitude summer insolation serves as the prevailing control on rainfall in the early Pleistocene at 20° N (112), possibly related to greater sensitivity of the eastern African monsoon to seasonal/interannual climate events in the Indian Ocean basin (30). The precession-scale, insolation-driven control on early Pleistocene climate contrasts with the role of ice-volume fluctuations and high-latitude climate change as a dominant influence on eastern African rainfall since 130 ka (112).

### Early Pleistocene Environment of Paleolake Olduvai.

The *δ*^13^C_org_ profile reflects shifts in the source of sedimentary OM from terrestrial vegetation to aquatic producers associated with decreasing rainfall and runoff, which is also recorded by evidence of changes in microbial utilization of OM (Figs. 3*B* and 4*B*). Evidence from leaf plant wax signatures portrays a landscape throughout Upper Bed I characterized by long-term (~40 ky) temporal stability in the proportions of C_3_ and C_4_ vegetation, with the latter more prominent (Figs. 3*A* and 4*A*). The absence of evidence for variations in the relative proportions of C_3_ and C_4_ plants throughout precession-driven wetter and drier intervals (Figs. 3*A* and 4*A*) contrasts with interpretations from prior investigations of time-equivalent outcrop samples from Location 80 [Fig. 1*A*, (7)] and refute the idea of significant changes in terrestrial vegetation in the Olduvai region associated with changes in climate driven by precession cycles. Thus, resources associated with terrestrial vegetation variously utilized by different hominin genera (113–115) likely remained stable during changes in hydroclimate, comparable with the temporal uniformity in the diet of *Homo* (115). It is therefore possible that other factors affecting paleolandscapes may have exerted a more significant influence on hominin evolution by inducing environmental stresses on key microhabitats. For example, evidence for wildfires is provided by the presence of charcoal and the distributions of pyrogenic polycyclic aromatic hydrocarbons (PAH) in Upper Bed I. Pyrogenic PAH in OGCP Core 2A sediments occur in higher abundance during wetter intervals and are attributed to increased fuel supply from terrestrial vegetation (116), comparable to interpretation of the microcharcoal record for Lake Turkana (117).

In contrast with their minimal effect on the balance of C_3_ vs. C_4_ vegetation, the wet/dry cycles produced substantive changes in lake dynamics, including increased lake alkalinity during drier intervals recorded in the *δ*^13^C values for biomarkers derived from aquatic plants and algae (Figs. 3*B* and 4*B*). Assessment of these biomarker characteristics, especially when morphological remains of aquatic biota are lacking as in Upper Bed I of OGCP Core 2A (10), can provide insights of climatic effects that are not recorded by proxies for terrestrial vegetation. Thus, temporal fluctuations in aquatic resources associated with wet/dry cycles likely represent the dominant climatic effect on the environmental landscape. In this scenario the region of Paleolake Olduvai did not experience shifts in the proportion of C_3_ and C_4_ vegetation during episodes of high climate variability (118). The inferred stability in savanna vegetation over ~40 ky likely fostered a sustained ecology for grazers and browsers, engendering hominin adaptations in response to this temporal consistency in animal communities, aligned with evidence for the gradual and continuous evolution of mammals in eastern Africa (119). Profound changes in lake size and alkalinity occurring over 0.5 to 1.8 ky associated with wet/dry climate transitions would have affected hominin access to food and water resources. For example, biomarker evidence for variations in the abundance of macrophytes may have impacted *Paranthropus bosei* whose diet included sedges (113). The high alkalinity of Paleolake Olduvai during drier episodes lasting 5 to 7 ky may also have increased hominin reliance on local freshwater springs (57, 120, 121). Indeed, changes in water resources rather than in terrestrial ecology may represent the major stressor for hominins during deposition of Upper Bed I.

Our study provides evidence of temporal features of vegetation and lake chemistry over ~40 ky that help frame the environmental setting for hominin activity well represented by fossil and tool records from Olduvai Gorge (Fig. 1). Our results confirm that terrestrial vegetation in the catchment of Paleolake Olduvai was consistent throughout wet/dry climate cycles while these hydroclimate shifts would have impacted local water resources. These insights derive from investigations of stratigraphic profiles for biomarkers derived from multiple sources (algae, bacteria, and land plants) and suggest that similar studies for other eastern African lakes would help determine whether the record for Paleolake Olduvai in the early Pleistocene is representative of regional ecological characteristics. Assessment of paleoclimate records at still higher temporal resolution for the Upper Bed I sediment cores deposited in Paleolake Olduvai could further constrain the sensitivity of this lake system to variations in effective moisture and climate forcing. Our study well demonstrates the advantages of examination of multiple molecular and isotopic proxies in sediment cores in efforts to decipher paleoenvironments and illustrates the potential limitations of low-resolution outcrop studies. It highlights the need to evaluate the combined influence of both terrestrial and aquatic sources of OM on *δ*^13^C_org_ profiles of lacustrine sediments, especially in alkaline lake systems, and confirms that combined molecular and isotope stratigraphy can serve as an invaluable tool in the reconstruction of paleolandscapes linked with hominins.

## Materials and Methods

OGCP cores recovered in 2014 were shipped to the National Lacustrine Core Facility (LacCore) at the University of Minnesota (now the Continental Scientific Drilling facility CSD) where they were split, processed, and described prior to long-term storage (*SI Appendix*). The Upper Bed I segment of Core 2A was sampled at ~16 cm intervals from the center of the working half of the core in efforts to reduce potential contamination from its exterior edges. One series of samples (~1 g) was used for bulk elemental and isotopic analyses, and a second series of 3 cm intervals (~20 g) was taken for detailed biomarker analyses (35, 45–48).

Biomarkers were extracted at 100 °C with CH_2_Cl_2_/MeOH (1:1 v:v) utilizing an Automated Solvent Extractor (45, 46). The resultant total lipid extracts were separated chromatographically into three fractions using activated Al_2_O_3_ columns, eluting sequentially with 4 mL of hexane/CH_2_Cl_2_ (9:1; v:v), hexane/CH_2_Cl_2_ (1:1; v:v), and CH_2_Cl_2_/MeOH (1:1 v:v) to yield fractions containing aliphatic hydrocarbons (F1), aromatic hydrocarbons and ketones (F2), and polar lipids (F3), respectively. Biomarker compositions for F1 and F2 fractions were determined using gas chromatography-mass spectrometry (46). This study utilizes F1 and F2 fractions whose biomarker compositions were previously reported (45–48, 51). The fractions were dried under N_2_ and resuspended in hexane (50 to 300 µL, depending on compound concentrations) for *δ*^13^C compound-specific isotopic analyses (CSIA) of individual biomarkers using a Trace Ultra gas chromatograph connected via a combustion reactor to a Thermo Delta V isotope ratio mass spectrometer (*SI Appendix*). Determination of *δ*^13^C values employed *n*-alkane standard mixtures in the calibration of biomarker data (*SI Appendix*, Table S2), which reports components that were present in concentrations sufficient to yield strong signals and also well-resolved from other constituents.

## Supplementary Material

Appendix 01 (PDF)

Dataset S01 (XLSX)

## Data Availability

All study data are included in the article and/or supporting information. Previously published data were used for this work. [All isotope data (d13Corg and d13C for all biomarkers) are included in *SI Appendix*, Table S1. Data on the distributions and abundances of biomarkers were previously published (34, 45–48, 51), but the d13C biomarker data is new and unpublished.]
