# Supplementary material for: Biomarker δ13C values record consistent savanna vegetation and variable alkalinity of Lake Olduvai during Pleistocene wet/dry cycles
Source: Proc Natl Acad Sci U S A. 2025 Dec 29;123(1):e2508896122. doi: 10.1073/pnas.2508896122 (PMC12773765; doi:10.1073/pnas.2508896122)
Supplement: Supplementary file 1 — Appendix 01 (PDF) [file pnas.2508896122.sapp.pdf]

PNAS

# Supporting Information for

## Biomarker $\delta^{13}C$ values record consistent savanna vegetation and variable alkalinity of Lake Olduvai during Pleistocene wet/dry cycles

Kelsey E. Doiron[a*], Devon E. Colcord[a], Andrea M. Shilling[a], Jackson K. Njau[a], Ian G. Stanistreet[b,d], Harald Stollhofen[c], Kathy D. Schick[d], Nick Toth[d], Simon C. Brassell[a]

*Kelsey E. Doiron, Simon C. Brassell.
**Email:** kelsey.doiron@eas.harvard.edu, simon@iu.edu

**This PDF file includes:**

Supporting text
Figure S1
Figure S2
Figure S3
Tables S1 and S2
Legend for Dataset S1
SI References

**Other supporting materials for this manuscript include the following:**

Dataset S1

## Supporting Information Text

### Chronology of Upper Bed I, Olduvai Gorge

Three critical marker beds, designated as Bed I Basalt (Fig. 1B), Tuff IB, and Tuff IF, have long been established from studies of outcrops in Olduvai Gorge (1-4). They provide chronological control (5) of the Bed I stratigraphic succession recovered at OGCP Core 2A, further aided by lithostratigraphic correlations (6) and geochemical fingerprinting (7). $^{40}Ar/^{39}Ar$ dating of samples from OGCP Core 2A (8) confirmed and refined the age of Tuff IB (1.8479 ± 0.0027 Ma), comparable to previous reports (1.848 ± 0.003 Ma (5)). However, the Bed I basalt age, previously thought to be 1.877 ± 0.013 Ma (5) was revised to an earlier age (1.900 ± 0.015 Ma; (6,8)). This age correction revises the extrapolated sedimentation rates for Upper Bed I of Core 2A to ~0.36 cm/yr (9), which corresponds to a resolution of ~571 yr for samples taken at 16 cm intervals, with each 3 cm subsampled interval spanning ~107 years of sediment deposition. These age assessments align with estimates of the number of laminae (~30 per cm) within the W1 interval containing laminated claystones. However, the presence of desiccation cracks, animal footprints, and grassland vegetation in lake sediments at several levels within the stratigraphic section confirms the presence of hiatuses (10-13) within the sequence, which become more pronounced higher in the section associated with Tuff IF (6). These hiatuses mean that the sediment accumulation rate of ~0.36 $mmyr^{-1}$, which is interpolated from the ages and core depths of the Bed I basalt (1.900 at 90.3 mbs) to Tuff IB (1.848 at 72 mbs), represents a minimum value (14). The duration of specific intervals for Upper Bed I corresponding to transitions in the $\delta^{13}C_{org}$ profile (Fig. 2) were calculated using interpolated ages for relevant depths based on $^{40}Ar/^{39}Ar$ dates for the Bed I basalt (1.900 Ma) and Tuff IB (1.848 Ma) in Core 2A (8) and assuming a uniform sediment accumulation rate (**SI Table 1**). The transitions themselves represent intervals between samples exhibiting substantive differences in their $\delta^{13}C_{org}$ of ~6 ‰ to 11 ‰.

### Core descriptions and sampling

In 2014, the OGCP was successful in retrieving ~245 meters of sediment core from Borehole 2A (02° 58' 43.0" S, 035° 19' 25.5" E; Fig. 1A), with a recovery rate of approximately 94 %. This location was specifically chosen based on decades of investigations of the geology of Olduvai Gorge, coupled with data from geophysical surveys in order to target recovery of the thickest stratigraphic sequence associated with the proposed depocenter of Paleolake Olduvai (1,15,16). The depocenter of the lake was controlled by faults and attained its greatest extent and depth during the wetter intervals of Upper Bed I (6,17). However, the presence of multiple erosion surfaces within Upper Bed I in Core 2A (Fig, 1) attests to dynamic changes in lake levels associated with changes in precipitation and run-off linked to precession cycles, likely augmented by tectonic controls on topography (13,17,18). Care was exercised during the drilling operations and subsequent handling of the cores recovered to avoid sediment contamination by lubricants or other oil-based substances. Upon retrieval, each core segment was encased in a tube, secured, and transported to the National Lacustrine Core Facility (LacCore) at the University of Minnesota for Initial Core Descriptions, sampling, and long-term cold storage. After splitting and processing of each core, two series of samples were collected at ~16 cm intervals from the center of the working half of the Upper Bed I segment of Core 2A in efforts to reduce potential contamination from the outer edges of the core. The first series (~1 g) of samples were gathered for bulk elemental and isotopic geochemical analyses (**SI Dataset**). Concurrently, the second series of 3 cm core section samples (~20 g) were collected for detailed biomarker analyses (9,13,14,18,19). Most critically, these cores afforded the opportunity to conduct continuous sampling of unweathered sediments at higher stratigraphic resolution than possible from outcrops and provide better preservation of their OM and constituent biomarkers.

### $\delta^{13}$Corg and C/N ratios

The profiles of $\delta^{13}C_{org}$ and C/N values for Bed I have been previously reported (12,13,16) and show systematic differences between wetter and drier intervals (**SI Dataset**). A cross-plot of these data sets complemented by those for the transitions between them and the interval between tuffs IB and IF allows comparison with the established ranges for $C_3$ and $C_4$ land plants and lacustrine algae (20; **SI Fig. 1**). It reveals that these separate data sets are clustered within discrete regions of the graph. Differences in C/N ratios for the samples mean that the drier intervals plot closer to the field for $C_4$ plants (20), whereas the area for samples from wetter intervals extends into the field for $C_3$ plants (20). Samples from the transitional intervals plot in the intermediate space and those from the interval between Tuffs IB and IF, which is an interval with evidence for increasing desiccation (9), plot toward the field for lacustrine algae (20).

## $\delta^{13}$C Determination of Biomarkers

$\delta^{13}$C compound-specific isotopic analyses (CSIA) of individual biomarkers employed a Trace Ultra gas chromatography connected via a combustion reactor to a Thermo Delta V isotope ratio mass spectrometer in the Indiana University Stable Isotope Research Facility. Compounds were separated with cool-on-column injections on a Trace Ultra GC equipped with a DB-5 column (30 m x 0.25 mm i.d.) using a He flow rate of 1 mL $min^{-1}$. The oxidation temperature for the reactor containing a combination of different metals (i.e., Ni, Pt, Cu) was set to 940 °C.

*n*-Alkane standard mixtures were used for calibration of components, yielding the isotopic values compiled in **SI Table 2**. The table reports data for individual compounds present in sample fractions in sufficient concentrations for measurement of their $\delta^{13}$C signals in instances where their peaks were separated and resolved from other constituents. The low concentration of specific compounds in some samples (9,13,14,18), and/or their coelution with other constituents, precluded determination of a full suites of the target compounds in every sample.

The standard mixture was analyzed at the beginning and end of each analysis day and a subset of samples were analyzed for replicates. No peak area effect was observed when samples and standards were measured at different concentrations. Carbon isotopic compositions were normalized to Vienna Pee Dee Belemnite (VPDB) using a mixture of *n*-alkanes ($C_{16}$ through $C_{30}$) of known isotopic composition (mixtures B5 and C5 prepared by Dr. Arndt Schimmelmann). The $\delta^{13}$C values for individual *n*-alkanes in the standard are presented in **SI Table 2**.

The temperature program for gas chromatography in GC-IRMS analyses employed a temperature ramp of 2 °C/min to ensure that the peaks for hop-17(21)-ene and the *n*-$C_{31}$ alkane were well separated. Faster ramps (e.g., 4 °C/min) led to the co-elution of these components leading to an aggregate, invalid $\delta^{13}$C value. For dry intervals the aggregate $\delta^{13}$C for a coeluting mix of hop-17(21)-ene (avg. -20.07 ‰) and *n*-$C_{31}$ (avg. -23.42 ‰; **SI Dataset**) would be skewed to intermediate values dependent on the relative proportions of the two compounds and might therefore be interpreted to reflect an increase in $C_4$ contributions. Use of urea adduction or 5Å molecular sieves to separate *n*-alkanes from branched and cyclic aliphatic hydrocarbons, as previously reported (21), eliminates this possibility.

## Statistical Assessment of Relationships among $\delta^{13}C_{org}$ and Biomarker $\delta^{13}$C Values

The correlation of $\delta^{13}C_{org}$ and $\delta^{13}C_{\Delta17(21)H}$ for all samples and for the D1 through W2 interval show an enhanced correlation for the latter (**SI Fig. 2**). ANOVA tests of relationships among $\delta^{13}$C values for individual *n*-alkanes and for each category of source-diagnostic biomarkers, including comparisons of wet versus dry cycles (**SI Dataset**; **Fig. 3**) were determined (**SI Fig. 3**). They reveal no significant differences in the $\delta^{13}$C compositions for: (i) several plant wax *n*-alkanes, including aggregate values for wet versus dry cycles, (ii) $\delta^{13}C_{org}$ and $\delta^{13}C_{\Delta17(21)H}$ for the D1 through W2 interval, and (iii) homohopane ($C_{31}$H) and sterenes across wet/dry cycles. The ANOVA tests confirm differences in the $\delta^{13}$C profiles of plant waxes compared with those of aquatic macrophytes, algae (sterenes and alkenones), and bacteria (hopanoids).

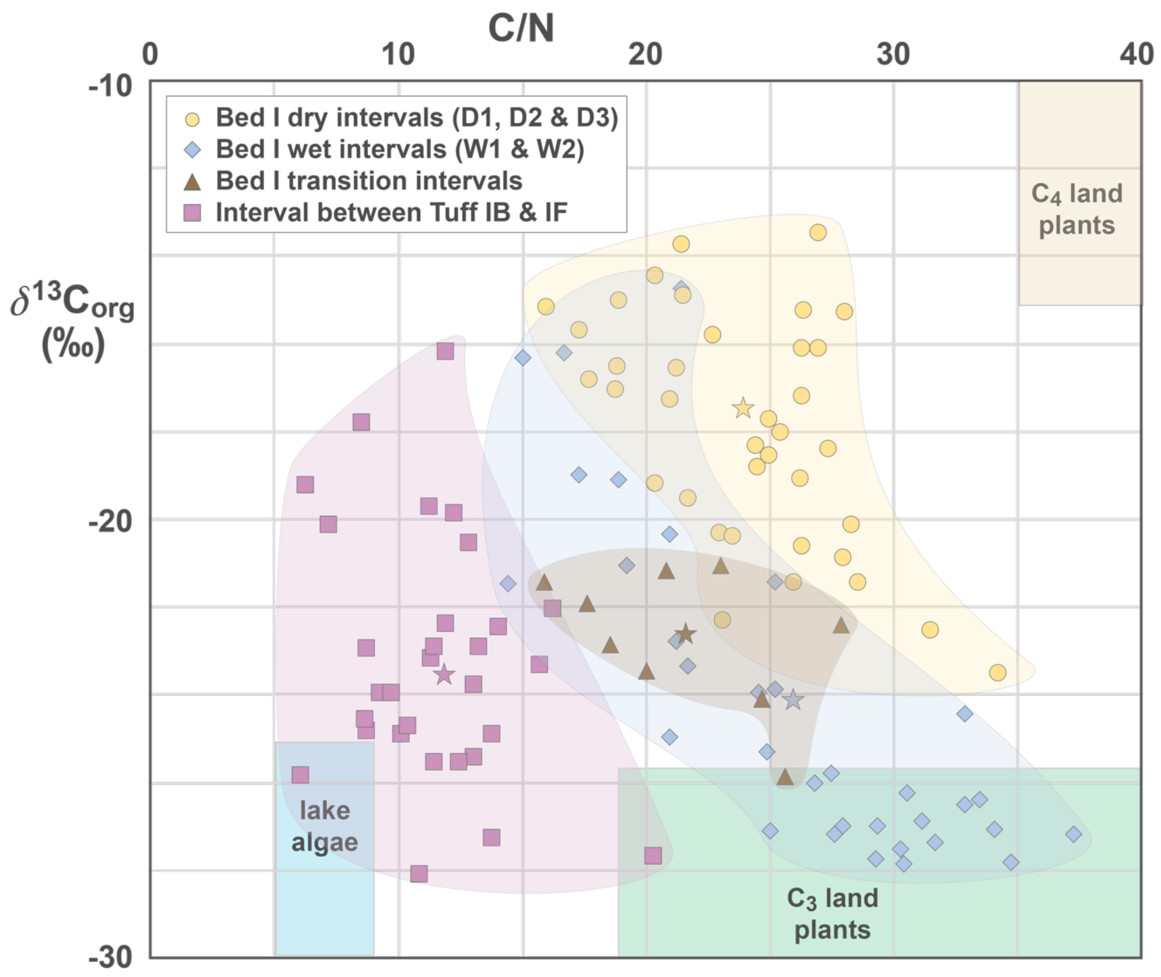


**Fig. S1**. A cross plot of C/N ratios and $\delta^{13}C_{org}$ (‰) for Bed I (**SI Dataset**) illustrating the different fields of the graph occupied by samples from dry and wet stratigraphic intervals, the transitions between them (Fig. 2B), and the interval between Tuffs IB (72 mbs) and IF (64 mbs). The average values for each of the four groups is represented by a star. The range of values associated with $C_3$ and $C_4$ land plants and lacustrine algae (20) are also shown.

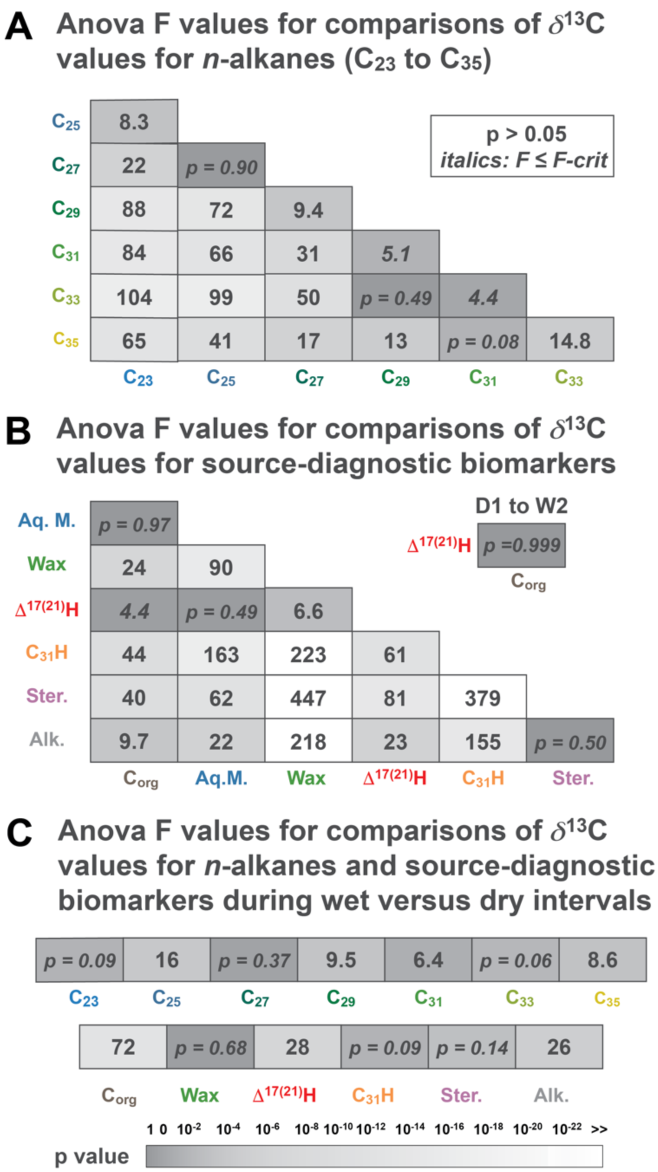


**Fig. S2.** Statistical evaluation of relationships among $\delta^{13}$C data (**SI Dataset**) for Bed I. **A.** Intercomparison of $\delta^{13}$C values for *n*-alkanes ($C_{23}$, $C_{25}$, $C_{27}$, $C_{29}$, $C_{31}$, $C_{33}$, $C_{35}$) illustrating similarity between components derived from aquatic macrophytes and land plants. **B.** Intercomparison of $\delta^{13}$C data for $C_{org}$ and source diagnostic biomarkers (Aq.M. = aquatic macrophyte *n*-alkanes ($C_{23}$ & $C_{25}$), Wax = land plants *n*-alkanes ($C_{29}$, $C_{31}$, $C_{33}$ & $C_{35}$), $\Delta^{17(21)}$H = hop-17(21)-ene, $C_{31}$H = 17β(H),21β(H)-homohopane, Ster. = algal sterenes, and Alk. = alkenones). **C.** Intercomparison of $\delta^{13}$C data for *n*-alkanes, $C_{org}$ and source diagnostic biomarkers for drier and wetter intervals.

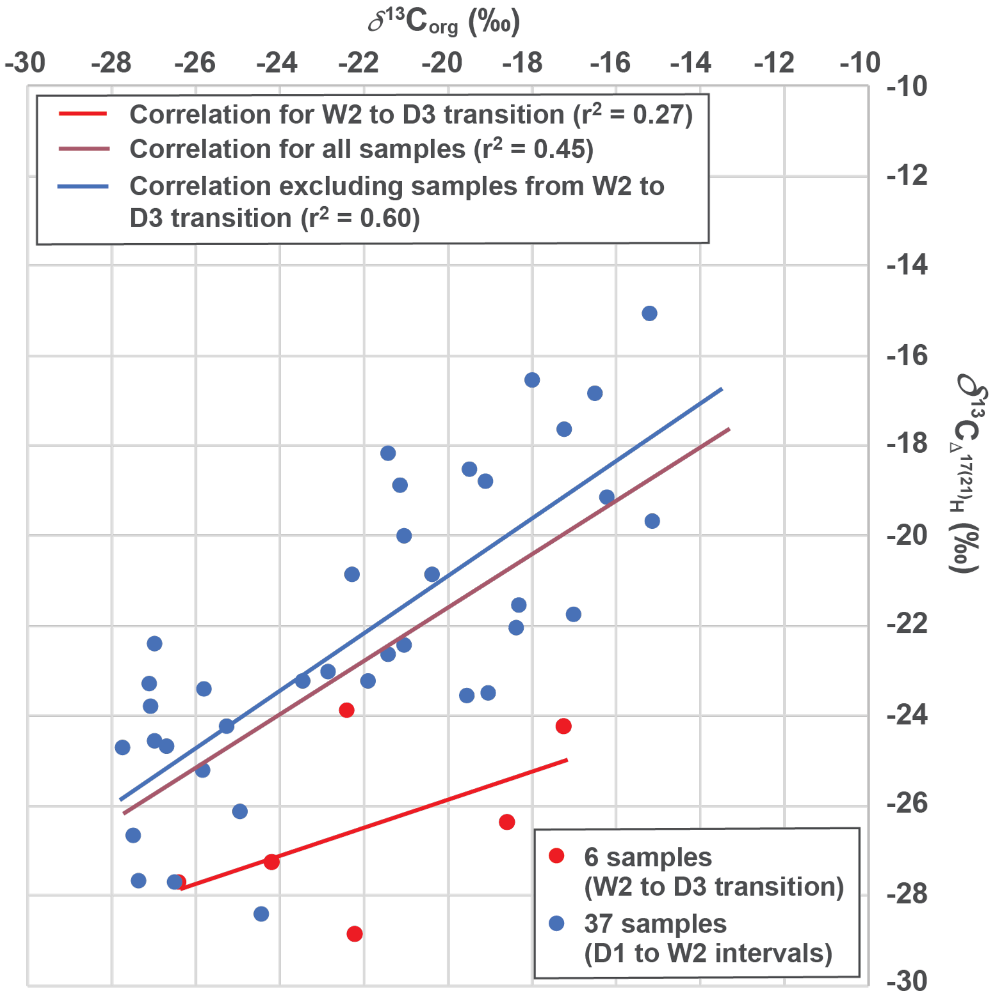


**Fig. S3.** Correlations between $\delta^{13}C_{org}$ and $\delta^{13}C$ for hop-17(21)-ene ($\delta^{13}C_{\Delta 17(21)H}$) for all samples, for the W2 to D3 transition, and for D1 through W2 up to the transition to D3.

**Table S1. Determination of the approximate duration of drier and wetter intervals in Core 2A, and their transitions (Fig. 2C), based on interpolated ages.**

| Horizon | Depth (mbs) | Thickness (m) | Interpolated Age (Ma) | Duration (yr) |
|---|---|---|---|---|
| ***Tuff IB at 72.0 mbs with an $^{40}Ar/^{39}Ar$ date of 1.848 ± 0.003 (5,8)*** | | | | |
| D3 | | 2.45 | | ~7,000 |
| Base D3 | 74.45 | | 1.8550 | |
| Transition | | 0.30 | | ~850 |
| Top W2 | 74.75 | | 1.8558 | |
| W2 | | 4.15 | | ~11,800 |
| Base W2 | 78.90 | | 1.8676 | |
| Transition | | 0.30 | | ~850 |
| Top D2 | 79.20 | | 1.8685 | |
| D2 | | 2.38 | | ~6,750 |
| Base D2 | 81.58 | | 1.8752 | |
| Transition | | 0.64 | | ~1,800 |
| Top W1 | 82.22 | | 1.8770 | |
| W1 | | 2.79 | | ~7,900 |
| Base W1 | 85.01 | | 1.8850 | |
| Transition | | 0.15 | | ~450 |
| Top D1 | 85.16 | | 1.8854 | |
| D1 | 86.87 | 1.71 | | ~ 4,800 |
| base D1 | 89.84 | 4.68 | 1.8987 | ~13,300 |
| ***Bed I Basalt at 90.3 mbs with an $^{40}Ar/^{39}Ar$ date of 1.900 ± 0.015 (8)*** | | | | |

**Table S2. $\delta^{13}C$ values for individual *n*-alkanes in the B5 and C5 standard mixtures**

**B5 *n*-alkane standard mixture**

| ***n*-Alkane** | **$\delta^{13}C$ (‰)** | ***n*-Alkane** | **$\delta^{13}C$ (‰)** | ***n*-Alkane** | **$\delta^{13}C$ (‰)** | ***n*-Alkane** | **$\delta^{13}C$ (‰)** |
|---|---|---|---|---|---|---|---|
| 16 | -30.66 ± 0.02 | 17 | -31.16±0.02 | 18 | -31.10±0.01 | 19 | -33.17±0.01 |
| 20 | -32.35±0.02 | 21 | -29.10±0.02 | 22 | -32.87±0.02 | 23 | -31.77±0.02 |
| 24 | -33.34±0.02 | 25 | -28.48±0.02 | 26 | -33.03±0.01 | 27 | -29.56±0.01 |
| 28 | -32.21±0.01 | 29 | -30.07±0.02 | 30 | -29.86±0.02 | | |

**C5 *n*-alkane standard mixture**

| ***n*-Alkane** | **$\delta^{13}C$ (‰)** | ***n*-Alkane** | **$\delta^{13}C$ (‰)** | ***n*-Alkane** | **$\delta^{13}C$ (‰)** | ***n*-Alkane** | **$\delta^{13}C$ (‰)** |
|---|---|---|---|---|---|---|---|
| 17 | -31.88±0.01 | 19 | -31.99±0.01 | 21 | -28.83±0.02 | 23 | -33.37±0.03 |
| 25 | -28.46±0.02 | | | | | | |

*Reference*: https://hcnisotopes.earth.indiana.edu/reference-materials/index.html

The root mean square error of replicate measurements of standards during the sequence of $\delta^{13}C$ analyses averaged ±0.3 ‰.

**Dataset S1 (separate file).**

**Bulk geochemical data and $\delta^{13}C$ biomarker compositions for Olduvai Core 2A samples**

**SI References**


1. R. L. Hay, *Geology of the Olduvai Gorge: A Study of Sedimentation in a Semi Arid Basin*, University of California Press, 203 pp. (1976).
2. H. Stollhofen *et al*., Fingerprinting facies of the Tuff IF marker, with implications for early hominin palaeoecology, Olduvai Gorge, Tanzania. *Palaeogeog., Palaeoclimat., Palaeoecol.*, *259*, 382–409 (2008).
3. L. J. McHenry, A revised stratigraphic framework for Olduvai Gorge Bed I based on tuff geochemistry. *J. Human Evol.*, *63*, 284–299 (2012).
4. J. M. Habermann *et al*., (2016). Discrimination, correlation, and provenance of Bed I tephrostratigraphic markers, Olduvai Gorge, Tanzania, based on multivariate analyses of phenocryst compositions. *Sed. Geol.*, *339*, 115–133 (2016).
5. A. L. Deino, $^{40}Ar/^{39}Ar$ dating of Bed I, Olduvai Gorge, Tanzania, and the chronology of early Pleistocene climate change. *J. Human Evol.*, *63*, 251–273 (2012).
6. I. G. Stanistreet *et al*., New Olduvai Basin stratigraphy and stratigraphic concepts revealed by OGCP cores into the Palaeolake Olduvai depocentre, Tanzania. *Palaeogeog., Palaeoclimat., Palaeoecol.*, *554*, 109751 (2020).
7. L. J. McHenry *et al*., Lake conditions and detrital sources of Paleolake Olduvai, Tanzania, reconstructed using X-ray Diffraction analysis of cores. *Palaeogeog., Palaeoclimat., Palaeoecol.*, *556*, 109855 (2020).
8. A. L. Deino *et al*., Chronostratigraphy and age modeling of Pleistocene drill cores from the Olduvai Basin, Tanzania (Olduvai Gorge Coring Project). *Palaeogeog., Palaeoclimat., Palaeoecol.*, *571*, 109990 (2021).
9. A. M. Shilling *et al*., Biogeochemical evidence from OGCP Core 2A sediments for environmental changes preceding deposition of Tuff IB and climatic transitions in Upper Bed I of the Olduvai Basin. *Palaeogeog., Palaeoclimat., Palaeoecol.*, *555*, 109824 (2020).
10. H. Stollhofen *et al*., Fingerprinting facies of the Tuff IF marker, with implications for early hominin palaeoecology, Olduvai Gorge, Tanzania. *Palaeogeog., Palaeoclimat., Palaeoecol.*, *259*, 382–409 (2008).
11. M. K. Bamford, I. G. Stanistreet, H. Stollhofen, R. M. Albert, Late Pliocene grassland from Olduvai Gorge, Tanzania. *Palaeogeog., Palaeoclimat., Palaeoecol.*, *257*, 280–293 (2008).
12. D. E. Colcord *et al*., Aquatic biomarkers record Pleistocene environmental changes at Paleolake Olduvai, Tanzania. *Palaeogeog., Palaeoclimat., Palaeoecol.*, *524*, 250–261 (2019).
13. A. M. Shilling *et al*., Biogeochemical evidence for environmental changes of Pleistocene Lake Olduvai during the transitional sequence of OGCP core 2A that encompasses Tuff IB (~1.848 Ma). *Palaeogeog., Palaeoclimat., Palaeoecol.*, *532*, 109267 (2019).
14. T. M. Ferland *et al*., Biased preservation of Pleistocene climate variability proxies at Olduvai Gorge, Tanzania. *Palaeogeog., Palaeoclimat., Palaeoecol.*, *562*, 109940 (2021).
15. I. G. Stanistreet, Fine resolution of early hominin time, Beds I and II, Olduvai Gorge, Tanzania. *J. Human Evol.*, *63*, 300–308 (2012).
16. J. K. Njau *et al*., The Olduvai Gorge Coring Project: Drilling high resolution palaeoclimatic and palaeoenvironmental archives to constrain hominin evolution. *Palaeogeog., Palaeoclimat., Palaeoecol.*, *561*, 110059 (2021).
17. I. G. Stanistreet *et al*., Changing depocentre environments of Palaeolake Olduvai and carbonates as marker horizons for hiatuses and lake-level extremes. *Palaeogeog., Palaeoclimat., Palaeoecol.*, 560, 110032 (2020).
18. D. E. Colcord *et al*., Sub-Milankovitch paleoclimatic and paleoenvironmental variability in East Africa recorded by Pleistocene lacustrine sediments from Olduvai Gorge, Tanzania. *Palaeogeog., Palaeoclimat., Palaeoecol.*, *495*, 284–291 (2018).
19. S. C. Brassell *et al*., Alkenones in Pleistocene Upper Bed I (1.803–1.900 Ma) sediments from Paleolake Olduvai, Tanzania. *Org. Geochem.*, *170*, 104437 (2022).
20. P. A. Meyers, Applications of organic geochemistry to paleolimnological reconstructions: a summary of examples from the Laurentian Great Lakes. *Org. Geochem.*, *34*, 261-289 (2003)
21. C. R. Magill, G. M. Ashley, K. H. Freeman, Ecosystem variability and early human habitats in eastern Africa. *PNAS*, *110*, 1167–1174 (2013).
